# Cytotoxicity Potentials of Eleven Bangladeshi Medicinal Plants

**DOI:** 10.1155/2014/913127

**Published:** 2014-11-09

**Authors:** Amina Khatun, Mahmudur Rahman, Tania Haque, Md. Mahfizur Rahman, Mahfuja Akter, Subarna Akter, Afrin Jhumur

**Affiliations:** ^1^Phytochemistry and Pharmacology Research Laboratory, Department of Pharmacy, School of Science, Engineering and Technology, Manarat International University, 1/B, 1/1, Zoo Road, Mirpur, Dhaka 1216, Bangladesh; ^2^Natural Product and Drug Discovery Lab, Department of Pharmacy, Faculty of Health Sciences, Northern University Bangladesh, 24 Mirpur Road, Dhaka 1205, Bangladesh; ^3^Product Management Department, The IBN Sina Pharmaceutical Industry Ltd., House No. 41, Road No. 10/A, Dhanmondi R/A, Dhaka 1205, Bangladesh

## Abstract

Various forms of cancer are rising all over the world, requiring newer therapy. The quest of anticancer drugs both from natural and synthetic sources is the demand of time. In this study, fourteen extracts of different parts of eleven Bangladeshi medicinal plants which have been traditionally used for the treatment of different types of carcinoma, tumor, leprosy, and diseases associated with cancer were evaluated for their cytotoxicity for the first time. Extraction was conceded using methanol. Phytochemical groups like reducing sugars, tannins, saponins, steroids, gums, flavonoids, and alkaloids were tested using standard chromogenic reagents. Plants were evaluated for cytotoxicity by brine shrimp lethality bioassay using *Artemia salina* comparing with standard anticancer drug vincristine sulphate. All the extracts showed potent to moderate cytotoxicity ranging from LC_50_ 2 to 115 *µ*g/mL. The highest toxicity was shown by *Hygrophila spinosa* seeds (LC_50_ = 2.93 *µ*g/mL) and the lowest by *Litsea glutinosa* leaves (LC_50_ = 114.71 *µ*g/mL) in comparison with standard vincristine sulphate (LC_50_ = 2.04 *µ*g/mL). Among the plants, the plants traditionally used in different cancer and microbial treatments showed highest cytotoxicity. The results support their ethnomedicinal uses and require advanced investigation to elucidate responsible compounds as well as their mode of action.

## 1. Introduction

Nature is the source of 87% of drugs used to treat all categorized human diseases. 25% of prescribed drugs originated from plants. Over 3000 species of plants have been reported for their anticancer property. Uddin et al. [1] stated that till now about 80% people in developing countries rely on traditional plant based medicines for their primary health care. Focus on natural products is increasing day by day as it serves as an enormous source of new anticancer drugs. According to Washart [2] natural products are vital in the treatment of cancer, as a number of important anticancer agents have been derived from natural products, including plant-derived agents such as the vinca alkaloids, taxanes, and topoisomerase I inhibitors. According to intercontinental marketing services report [3], the anticancer drug market in Bangladesh is growing at 20 percent a year due to an alarming rise in cancer patients. So, search for new anticancer drugs is the demand of time.

Bangladesh has a rich and prestigious heritage of herbal medicines among the South Asian countries. Ghani [4] estimated that about 250 species of medicinal plants are used for the preparation of traditional medicines which is the half of total species of plants grown in Bangladesh. However, the majority of these plants have not yet undergone chemical, pharmacological, and toxicological studies to investigate their bioactive compound(s). Traditional resources and ecological diversity indicate that Bangladeshi plants represent an exciting resource for new drug discovery.

Ahmed and Uddin [5, 6] identified* Melastoma malabathricum* L., synonym* M. polyanthum*, as a spreading shrub; leaves are opposite and lanceolate; flowers are solitary clustered, purple in color. The plant grows in the hill tracts and also in roadside of central and east region of Bangladesh. It is also found in Thailand, India, and Sri Lanka. Many researchers [7–10] recognized this plant as an ethnomedicinal plant in various countries of the world.* M. malabathricum* is reported as antiviral by Nazlina et al. [11], antibacterial by Choudhury et al. [12], antioxidant and cytotoxic by Alwash et al. [13], antinociceptive, anti-inflammatory, and antipyretic by Zakaria et al. [14], antidiarrhoeal by Sunilson et al. [15], antidiabetic and antihyperlipidemic by Kumar et al. [16], and hepatoprotective by Mamat et al. [17]. Sirat et al. [18] isolated amides, triterpenes, and flavonoids.

*Litsea glutinosa* (Lour.) Roxb., synonym* L. chinensis*, is medium-sized deciduous or semievergreen tree; leaves are aromatic, ovate, or elliptic. The plant grows in Bangladesh, India, Sri Lanka, Myanmar, China, and Malaysia as described by Uddin [6]. Mandal et al. [19] reported antimicrobial, Kar et al. [20] cardiovascular, and Menon et al. [21] psychopharmacological activity. Agrawal et al. [22] isolated four butenolides, Wang et al. [23] 2′-oxygenated flavone glycoside, and Herath et al. [24] D-xylose and L-arabinose from the plant.

*Malpighia coccigera* is not locally considered as a medicinal plant; rather it is an ornamental hedge plant. Seipold et al. [25] isolated floral oil from this plant. Evans [26] considered that other species of* Malpighia* are hallucinogenic.

Baker [27] identified* Pseudelephantopus spicatus* L. as a herb; hairs are present on both the upper and lower surfaces of the leaf blade, flowers are white borne in narrow bullet-shaped heads, four flowers per head, and the pappus is present on the end of each fruit. Odonne et al. [28] isolated three compounds: two hirsutinolides and ursolic acid having activity against* Leishmania amazonensis* from* P. spicatus*.

According to Uddin [29],* Viscum orientale* Willd., synonym* V. verticillatum* Roxb., is semiparasitic much-branched shrub on trees; leaves are opposite, thick, elliptic, or obovate, rarely lanceolate, obtuse, and glabrous, flowers are in axillary sessile or shortly pedunculate, acute, deciduous, and berry is ovoid or subglobose. The plant grows on various species of trees at low and medium altitudes forests of Chittagong, Chittagong hill tracts, and Sundarban mangrove forest in Bangladesh. It also occurs in India (Bihar, West Bengal, and Kerala). Schneeweiss [30] stated that the plant parasitizing* Strychnos nux-vomica* tree is used in Indian medicine. Satish et al. [31] reported the parasite for antibacterial activity. Lee et al. [32] evaluated another species,* V. album* for inhibitory activities of pancreatic lipase and phosphodiesterase. Park et al. [33] reported that* V. album* contains beta-galactoside- and N-acetyl-D-galactosamine-specific lectin II (60 kDa), polysaccharides, and viscotoxin (5 kDa) with their antitumor activity.

Naser et al. [34] described that* Thuja occidentalis* L. is indigenous to eastern North America and is grown in Europe as an ornamental tree with a maximal height. It has coniferous pyramidal features, with flattened branches and twigs in one plane, bearing small scale-like leaves. Over the whole year, the leaves are green, with the lower side showing a brighter green where resin glands also reside. Small coniferous pins contain the seeds. Kumar et al. [35] reported antibacterial, anticancer, anti-HIV, antispasmodic, antioxidant, antidiabetic, hepatoprotective, insecticidal, radioprotective, antiatherosclerosis, and neuropharmacological and Naser et al. [34] anti-influenza activities of this plant.

Ahmed [5] described* Hoya parasitica *(Roxb.), synonym* Asclepias parasitica*, as a tall climber; its stems are stout or slender, glabrous. Its leaves are ovate elliptic or lanceolate acute or acuminate peduncles are solitary or in pairs short or long slender or stout, pedicels slender long glabourous, coronal-processes longer than the corolla tube; the plant bears aesthetic flowers in May to June. It is mostly grown parasite on giant trees, found in the Chittagong, Sylhet, and Satkhira districts and in the Sundarbans of Bangladesh. It also grows in Assam, East Bengal, Tippera, Cronulla, Malacca, Singapore, and the Andaman Island. One of the same authors of this study [36, 37] reported the antibacterial and antinociceptive activities of* Hoya parasitica* leaves and growth inhibitory effects of dihydrocanaric acid against both HeLa and SW480 cells. Mukherjee et al. [38] reported the plant to contain triterpenic 3,4-seco acid 3,4-secolup-20(29)-en-3-oic acid, along with lupeol and lupenone from stem and Sadhu et al. [37] reported to contain an androstanoid, a sesquiterpene, and a phenolic compound, together with a known triterpene, dihydrocanaric acid.

Ahmed [5] stated that* Cnicus arvensis* (L.) Roth., synonym* Cirsium arvense* (L). Scop., is an erect herb; leaves are alternate; flower heads are solitary, hermaphrodite, pappus, and purple. It grows in sandy soils, river banks, and rice fields in Bangladesh. No scientific study has been reported yet.

According to Ghani, Ahmed, and Hooker Sir [4, 5, 39],* Commelina benghalensis *Linn. is a pubescent and ascending herb which grows all over Bangladesh. The plant is reported for its antimicrobial by Khan et al. [40], sedative and anxiolytic by Hasan et al. [41], and anticancer activity by Mbazima et al. [42].

*Baccaurea ramiflora* Lour., synonym* B. sapida* (Roxb.) Muell.-Arg., is described by Brandis [43]. It is a semievergreen tree; fruit is yellowish and velvety with pinkish white pulp. M. Sundriyal and R. C. Sundriyal [44] stated that it is native to Southeast Asia region. The plant has antioxidant property which was reported by Goyal et al. [45]. Yang et al. [46] isolated vanilloid derivatives from this plant.

Ahmed [5] described that* Hygrophila spinosa* T. Ander, synonym* H. auriculata*, is a stout, erect herb covered with stiff hair, leaves are opposite and lanceolate; flowers are in axillary whorls and purple. It grows throughout the plain districts of Indian subcontinent, in dump areas such as marshy margins of canals, rice fields, and so forth. It is also seen in tropical Himalaya, Ceylon, Myanmar, Indochina, and Malaya. Gomes et al. [47] reported the plant for its haematinic, Mazumdar et al. [48] for antitumor, and Kumari and Iyer [49] for diuretic activity. Previously any class of constituents was not reported. Folklore uses of these plants are summarized in Table 1.

## 2. Materials and Methods

### 2.1. Collection and Identification of Plant Material

The different parts of eleven plants were collected from different parts of Bangladesh. The samples of the plants were mounted on herbarium sheet and the species were taxonomically confirmed by Sarder Nasir Uddin, Principle Scientific Officer, Bangladesh National Herbarium (BNH), Mirpur, Dhaka. The voucher specimens of the plants have been deposited and preserved in BNH library for further collection and reference (Table 2).

### 2.2. Preparation of Methanol Extract

The collected different plant parts were separated from undesirable materials. They were dried in open air for two weeks. The shade dried plant parts were ground into a coarse powder with the help of a suitable grinder (capacitor start motor, Wuhu motor factory, China). The powders of the plant parts were stored in an airtight container and kept in a cool, dark, and dry place until the analysis commenced. Powered materials were taken in some clean, flat-bottomed glass containers and soaked in methanol. The containers along with their contents were sealed and kept for a period of 10 days with occasional shaking or stirring. The mixtures then underwent a coarse filtration by cotton and Whatman filter paper (Bibby RE200, Sterilin Ltd., UK). The filtrates were concentrated under air. Different amounts of concentrate extracts were obtained which were designated as crude methanol extracts (Table 3). Extraction was conceded following the method depicted by Khatun et al. [55].

### 2.3. Chemicals and Reagents

Standard chromogenic reagents lead acetate, potassium dichromate, ferric chloride, hydrochloric acid, sulfuric acid, Mayer's reagent, Dragendorff's reagent, Wagner's reagent, Hager's reagent, Molisch reagent, Benedict's reagent, and Fehling's solutions used for preliminary phytochemical chemical group test were of reagent grade and purchased from Sigma-Aldrich Co. LLC, Missouri, United States. Vincristine sulfate, used as a standard drug in the cytotoxic assay, was collected from the Techno Drugs Limited, Bangladesh. Methanol supplied by Laboratory Patterson Scientific, UK, was used as solvent for maceration of the plant material. Dimethyl sulfoxide (DMSO, ≥99.9%, BioReagent, for molecular biology; Sigma-Aldrich, India) was used to dissolve the extracts.

### 2.4. Instruments and Equipment

Electronic balance (serial number 1508, OHAUS, Germany) was used for this study. Glass-made hatching tank, air pump, and cover lampto grow shrimp were purchased locally. Pipettes, micropipette, test tubes, and other glass apparatus used were of laboratory standard and procured from authorized dealer.

### 2.5. Test for Different Chemical Groups

The preliminary phytochemical screening of the crude methanol extract was carried out by using standard chromogenic reagents; lead acetate, potassium dichromate, ferric chloride, hydrochloric acid, sulfuric acid, Mayer's reagent, Dragendorff's reagent, Wagner's reagent, Hager's reagent, Molisch reagent, Benedict's reagent, and Fehling's solutions were used to detect steroids, alkaloids, gums, flavonoids, saponins, tannins, and reducing sugars using standard protocol [55]. The colour intensity or the precipitate formation was used as analytical responses to these qualitative tests. 10% (w/v) solution of the extract in methanol was used for each of the above tests (Table 3).

### 2.6. Test for Cytotoxic Activity

Brine shrimp lethality bioassay was performed using the method of Meyer et al. [56]. Brine shrimp nauplii were obtained by hatching brine shrimp (*Artemia salina*) eggs (Carolina Biological Supply Company, Burlington, NC, USA) in artificial sea-water (3.8% NaCl solution) for 24 hrs. Dissolution of 30 mg of each extract was performed in 3 mL of artificial sea water containing 20% DMSO to give concentration of 10 *μ*g/*μ*L. From this solution 10, 20, 40, 80, 160, and 320 *μ*L were transferred to each 10 mL vial and using artificial sea-water the volume was adjusted to 10 mL by artificial sea water to give concentrations of compound of 10, 20, 40, 80, 160, and 320 *μ*g/mL, respectively. Brine shrimp nauplii were grown in these solutions and their mortality was observed after 24 h. The mortalities at 0 and 320 *μ*g/mL were excluded to get more accurate results except standard vincristine sulfate due to its high toxicity. The resulting data were transformed to probit analysis software (LdP Line software, USA) developed by Finney [57] for determination of LC_50_ values of the extracts. Artificial sea-water medium containing DMSO used for the analysis was employed as negative control. Vincristine sulfate was used as standard in this assay.

## 3. Results and Discussion

Fourteen extracts of eleven medicinal plants were evaluated for their cytotoxicity. Among them four extracts, aerial part of* Cnicus arvensis*,* Pseudelephantopus spicatus*, and* Viscum orientale*,* Thuja occidentalis* leaves, and* Hygrophila spinosa* seeds showed potent cytotoxicity LC_50_ ranging from 2 to 22 *μ*g/mL in comparison with standard vincristine sulfate (LC_50_ = 2.04 *μ*g/mL). Other plants also showed quite high cytotoxicity LC_50_ ranging from 21 to 115 *μ*g/mL (Table 4). the highest cytotoxicity was found in* H. spinosa* seeds (LC_50_ = 2.93 *μ*g/mL) and the lowest in* Litsea glutinosa* leaves (LC_50_ = 114.71 *μ*g/mL). We evaluated two plants of the family Asteraceae (*C. arvensis *and* P. spicatus*) and both of the plants showed significant cytotoxicity. Leaves of* T. occidentalis* showed significant cytotoxicity where the bark of this plant showed moderate cytotoxicity.* T. occidentalis, H. spinosa*, and* C. arvensis *are used ethnomedicinally in the treatment of cancer. In our investigation, we found the highest cytotoxicity of these plants. But* L. glutinosa* leaves showed the lowest cytotoxicity though it is traditionally used in treatment of tumor. On the other hand,* Malpighia coccigera* is not recognized as traditional medicinal plant but showed significant cytotoxicity (LC_50_ = 35.96 *μ*g/mL).* L. glutinosa* and* M. coccigera* may be tested for their cytotoxicity by other method to evaluate the present result.* Melastoma malabathricum*,* L. glutinosa*,* P. spicatus*,* Viscum orientale*, and* Commelina benghalensis *are traditionally reputed as either antimicrobial agent or poisonous plant. Cytotoxicity of* Melastoma malabathricum* was evaluated earlier in a process other than this by Alwash et al. [13] and anticancer activity of* Hoya parasitica* is previously reported by Sadhu et al. [37].

## 4. Conclusion

According to Sagar et al. [58], a master herbalist can advise on potential herbal treatments derived from centuries of traditional observations and advanced traditional medical systems such as Ayurveda. It will be imperative to develop a new model of modern pharmacology based on traditional pharmacognosy. Traditionally used plants in cancer treatments proved their efficacy in different pathway like inhibition of angiogenesis and metastasis, induction of apoptosis, and so forth. The plants showing significant cytotoxicity can be investigated for their bioactive compounds and their mode of action. Compounds may be isolated from plants showing significant cytotoxicity to identify the cytotoxic compounds and elucidate the possible mode of action using suitable technique. Most of the cytotoxic drugs possess serious adverse effect and their efficacy is unpredictable. New cytotoxic compounds found from these plants may present us a group of new well-tolerated anticancer and antimicrobial drugs.

## Figures and Tables

**Table 1:** Folklore uses of the studied eleven plants.

Species	Family	Traditional use
*Baccaurea ramiflora* Lour.	Euphorbiaceae	Uddin [6] stated that the plant is used in diarrhoea, flatulence, gastric ulcer, ureterolithiasis, and jaundice.

*Cnicus arvensis *(L.) Roth.	Asteraceae	Ahmed [5] indicated that the plant is used as cholagogic and diuretic. Uddin [6] wrote that traditionally it is used in the treatment of cirrhosis, diabetes, excessive menstruation, gout, hyperacidity (gastritis), liver cancer, jaundice, and scabies.

*Commelina benghalensis* Linn.	Commelinaceae	According to Ahmed [5], the plant has reputation to be used as antiseptic, demulcent, emollient, and refrigerant. Ghani [4] stated that it is also used in leprosy.

*Hoya parasitica *(Roxb.)	Asclepiadaceae	Ahmed [5] credited leaves of this plant to treat rheumatism.

*Hygrophila spinosa *T. Ander	Acanthaceae	Ahmed [5] described that the leaves, seeds, and roots are traditionally used as diuretic and also for jaundice, rheumatism, diseases of urogenital tract, and bladder stones. Joshi [50] and Kapoor and Mitra [51] reported that it is a reputed remedy for arthritis. It is also used as aphrodisiac, roborant, demulcent, and diuretic. The plant is useful in cancer and tubercular fistula and juice in anaemia. It is the source of locally used Ayurvedic, Unani, and indigenous drug preparations having anabolic-cum androgen-like activity.

*Litsea glutinosa* (Lour.) Roxb.	Lauraceae	Uddin [6] reported that the plant is used in the treatment of anklitis, asthma, bone fracture, tumor, leucorrhoea, hook worm infestation, rheumatoid arthritis, jaundice, epilepsy, liver disease, and dysentery.

*Malpighia coccigera *	Malpighiaceae	Not found.

*Melastoma malabathricum* L.	Melastomataceae	Kabir et al. [52] reported that this plant is used among the Tripura tribes in Bangladesh for the treatment of jaundice. Ahmed [5] pointed that the plant is used in diarrhoea, dysentery, wound healing, and skin diseases and Uddin [6] credited the plant for use in abdominal pain, sores in tongue, oedema, gynecological diseases, and snake bite.

*Pseudelephantopus spicatus* L.	Asteraceae	Odonne et al. [28] reported that the plant is used by the ethnic group from the Peruvian Amazonia in leishmaniasis.

*Thuja occidentalis* L.	Cupressaceae	According to Naser et al. [34] and Kumar et al. [35], in folk medicine, *T. occidentalis* has been used to treat bronchial catarrh, enuresis, cystitis, psoriasis, uterine carcinomas, amenorrhea, external fungal infections of the skin (ringworm and thrush), headache, scurvy prevention, eczema, anal or genital warts, and rheumatism. It is used as abortifacient, emmenagogue, vermifuge, diuretic, and digestive aid.

*Viscum orientale* Willd.	Loranthaceae	Stuar [53] the plant was considered poisonous in folklore medicine; in India used as a substitute for nux-vomica and used for pustular itches. Leaves are burned to ashes which are then mixed with sulphur and coconut oil and rubbed on the body. Poultice is used in neuralgia in Bangladesh. Nayak et al. [54] stated that the plant is used in giddiness and stiffness in Orissa, India.

**Table 2:** Botanical information of eleven medicinal plants of Bangladesh.

Species	Family	Local name	English name	Collection area	Collection time	Acc. number^a^
*Baccaurea ramiflora *	Euphorbiaceae	Latkan	Burmese grape	Narsingdi	August	DACB-35863
*Cnicus arvensis *	Asteraceae	Birhalkanta	Creeping Thistle	Khulna	March	DACB-31,544
*Commelina benghalensis *	Commelinaceae	Kanchire	Benghal dayflower	Dhaka	November	DACB-32,495
*Hoya parasitica *	Asclepiadaceae	Bayupriya	Wax flower	Satkhira	April	DACB-30,224
*Hygrophila spinosa *	Acanthaceae	Talmakhana	Marsh barbel	Satkhira	July	DACB-31257
*Litsea glutinosa *(Lour.) Roxb.	Lauraceae	Kukur chita	Bollygum	Munshiganj	May	DACB-25734
*Malpighia coccigera *	Malpighiaceae	Khoi phool	Dwarf Holly	Dhaka	January	DACB-37527
*Melastoma malabathricum* L.	Melastomataceae	Dantrasha	Malabar melastome	Savar, Dhaka	July	DACB-15161
*Pseudelephantopus spicatus* L.	Asteraceae	Kukur jihba	Dog's tongue	Satkhira	January	DACB-35,012
*Thuja occidentalis *	Cupressaceae	Thuja	White cedar	Dhaka	June	DACB-37930
*Viscum orientale* Willd.	Loranthaceae	Banda	Mistletoe	Satkhira mangrove	July	DACB-38174

^a^Accession number.

**Table 3:** Phytochemical evaluation of the fourteen extracts of eleven medicinal plants of Bangladesh.

Plants	Part used	% yield	Red. sug.^a^	Alk.^b^	Str.^c^	Tan.^d^	Gum	Flv.^e^	Sap.^f^
*Baccaurea ramiflora *	Leaves	5.00	−	+	+	+	+	−	−
*Baccaurea ramiflora *	Bark	3.00	+	+	−	+	−	+	+
*Cnicus arvensis *	Aerial part	3.17	ND^g^	ND	ND	ND	ND	ND	ND
*Commelina benghalensis *	Aerial part	3.00	ND	ND	ND	ND	ND	ND	ND
*Hoya parasitica *	Stem	3.08	+	+	+	+	−	+	−
*Hygrophila spinosa *	Seeds	12.0	+	+	+	−	+	+	+
*Litsea glutinosa *	Leaves	2.90	−	+	+	+	−	−	−
*Malpighia coccigera *	Leaves	4.92	−	+	+	−	−	+	−
*Melastoma malabathricum *	Leaves	9.09	+	+	+	+	+	−	−
*Melastoma malabathricum *	Stem	3.22	+	+	−	+	−	−	−
*Pseudelephantopus spicatus *	Aerial part	3.4	−	+	+	+	−	+	−
*Thuja occidentalis *	Leaves	2.57	−	−	−	+	−	−	−
*Thuja occidentalis *	Bark	5.25	−	+	+	+	−	+	+
*Viscum orientale* Willd.	Aerial part	4.35	+	+	−	+	−	+	−

^a^Reducing sugar, ^b^alkaloid, ^c^steroid, ^d^tannin, ^e^flavonoid, ^f^saponin, and ^g^not done.

**Table 4:** Cytotoxicity of the fourteen extracts of eleven plants.

Test sample	Concentration of extract (*µ*g/mL)							
	0	10	20	40	80	160	320	^ a^LC_50_
*Baccaurea ramiflora* bark	0	15	30	35	55	70	100	88.57
*Baccaurea ramiflora* leaves	0	10	25	45	50	65	80	96.76
*Cnicus arvensis* aerial part	0	35	55	75	80	90	100	5.54
*Commelina benghalensis* aerial part	0	30	45	55	70	90	95	40.04
*Hoya parasitica *stem	0	10	30	60	85	90	100	51.74
*Hygrophila spinosa* seed	0	45	55	75	85	100	100	2.93
*Litsea glutinosa* leaves	0	10	10	29	45	62	80	114.71
*Malpighia coccigera *	0	25	45	65	75	80	100	35.96
*Melastoma malabathricum *leaves	0	10	40	65	70	80	100	53.84
*Melastoma malabathricum *stems	0	25	45	50	60	90	100	52.71
*Pseudelephantopus spicatus* aerial part	0	35	55	65	75	85	100	14.90
*Thuja occidentalis* bark	0	30	40	50	50	60	90	86.38
*Thuja occidentalis *leaves	0	15	65	80	90	90	90	10.20
*Viscum orientale* aerial part	0	30	45	75	80	90	100	21.63
Vincristine sulphate	0	55	70	75	75	100	100	2.04

^a^Median lethal concentration.
